# Biomarker-based evaluation of aflatoxin B1 exposure in cattle

**DOI:** 10.14202/vetworld.2025.1297-1305

**Published:** 2025-05-25

**Authors:** Priyadharshini Ponnusamy, Umaya Suganthi Rajendran, Madhavan Gopalakrishnan Nair, Uma Sambath, Raja Kumar, Jacob Thanislass, Avinash Warundeo Lakkawar, Vijayalakshmi Padmanaban, Poobitha Subbarayan

**Affiliations:** 1 Department of Veterinary Pathology, Rajiv Gandhi Institute of Veterinary Education and Research, Puducherry, India; 2 Bioenergetics and Environmental Sciences Division, ICAR-National Institute of Animal Nutrition and Physiology, Bengaluru, India; 3 Centre for Translational Research, Rajiv Gandhi Institute of Veterinary Education and Research, Puducherry, India; 4 Department of Veterinary Medicine, Rajiv Gandhi Institute of Veterinary Education and Research, Puducherry, India

**Keywords:** aflatoxin B1, aflatoxin B1-albumin adduct, aflatoxin B1-DNA adduct, biomarkers, cattle, chronic exposure, enzyme-linked immunosorbent assay

## Abstract

**Background and Aim:**

Assessment of aflatoxin B1 (AFB1) exposure in cattle traditionally relies on feed analysis, which may not reflect chronic exposure or accurately indicate individual susceptibility. This study aimed to evaluate the utility of serum AFB1-albumin adducts and blood AFB1-DNA adducts as biomarkers for assessing individual chronic AFB1 exposure in cattle, irrespective of immediate feed contamination levels.

**Materials and Methods:**

Blood samples were collected from 53 crossbred cattle from farms, clinical veterinary cases, and slaughterhouses in Puducherry, India. Feed samples (n = 40) from farm and clinical cases were analyzed for aflatoxin contamination using two-dimensional thin-layer chromatography. AFB1 exposure was quantified by measuring serum AFB1-albumin adducts and blood AFB1-DNA adducts using an indirect enzyme-linked immunosorbent assay. In addition, a novel method was developed to synthesize the aflatoxin B1-formamidopyrimidine (AFB1-FAPy) adduct *in vitro* and the synthesized adduct was characterized to serve as a standard for DNA adduct quantification.

**Results:**

AFB1 was detected in 50% of feed samples, with 70% of positive samples exceeding the maximum permissible limit of 20 μg/kg. Despite variable feed contamination, serum AFB1-albumin and blood AFB1-DNA adducts were consistently detected across all animal categories. Median AFB1-albumin adduct levels were similar among farm (0.730 pg/mg), clinical (0.670 pg/mg), and slaughterhouse (0.770 pg/mg) cattle (p = 0.731). Median AFB1-DNA adduct levels were highest in slaughterhouse cattle (18.33 pmol/μg DNA), followed by farm (14.76 pmol/μg DNA) and clinical cases (7.47 pmol/μg DNA), although differences were not statistically significant (p = 0.328). No significant correlation was observed between feed contamination levels and biomarker concentrations, highlighting the chronic nature of AFB1 exposure.

**Conclusion:**

The consistent detection of AFB1-albumin and AFB1-DNA adducts in cattle, irrespective of detectable aflatoxin levels in feed, underscores the limitations of traditional feed analysis for monitoring chronic exposure. The novel synthesis and robust detection of AFB1-FAPy DNA adducts further enhance the reliability of these biomarkers. These biomarkers are minimally invasive, sensitive, and valuable for chronic aflatoxin exposure assessment, aiding proactive management strategies to safeguard animal health and public food safety.

## INTRODUCTION

Aflatoxins are among the most important of the 400 known mycotoxins and are primarily produced by *Aspergillus flavus* and *Aspergillus parasiticus*. They are potent carcinogens capable of damaging the liver and other critical organs, causing numerous health complications, including death in severe cases [1]. Among the different types of aflatoxins, aflatoxin B1 (AFB1) is considered the most toxic, exerting hepatotoxic, immunotoxic, mutagenic, carcinogenic, and teratogenic effects in humans and animals [2]. Aflatoxicosis, a common and significant health issue, results from consuming aflatoxin-contaminated feed. All animal species are susceptible to aflatoxicosis; however, sensitivity levels differ among species [3]. Cattle and other ruminants exhibit greater resistance to aflatoxicosis compared to monogastric animals and poultry. Following ingestion, AFB1 is rapidly absorbed in the intestines and transported to the liver, where hepatic mixed-function oxidases (cytochrome P450 enzymes) metabolize it into several metabolites, including AFB1-8, 9-exo-epoxide (AFBO), aflatoxin M1 (AFM1), aflatoxin Q1, and aflatoxin P1. Among these metabolites, AFBO is highly reactive and covalently binds to nucleic acids and proteins, forming AFB1-DNA and AFB1-albumin adducts, respectively. These adducts are primarily responsible for the toxic effects associated with aflatoxin, particularly hepatotoxicity and carcinogenicity. AFM1, a hydroxylated metabolite of AFB1 excreted in dairy cow milk, also poses significant public health risks [3].

Clinical signs of aflatoxicosis in cattle include anorexia, diarrhea, weight loss, and decreased milk production, leading to substantial economic losses for farmers [4]. Conventionally, aflatoxicosis is diagnosed by correlating clinical signs with serum biochemical alterations, detecting aflatoxins in feed, and observing characteristic gross and histopathological lesions, especially in cases involving mortality [4]. However, assessing aflatoxin exposure through feed analysis may not accurately reflect chronic or long-term exposure at the individual or population level, since it only indicates immediate contamination. Prior study by Kensler and Eaton [5] in experimental animals and humans has demonstrated the value of monitoring protein and DNA adducts as effective biomarkers for aflatoxin exposure. The measurement of aflatoxin metabolites and nucleic acid adducts in human urine, serum, and milk suggests that these biomarkers are rapidly excreted, indicating short-term exposure. Specifically, AFB1-guanine adducts in urine only reflect exposure within the preceding 24 h and thus have limited utility for evaluating long-term exposure [5]. Conversely, AFB1-albumin adducts has a relatively prolonged biological half-life compared to other biomarkers, making them a reliable indicator for chronic exposure. Although the persistence of AFB1-formamidopyrimidine (AFB1-FAPy) DNA adducts in liver tissues could serve as a useful biomarker for AFB1 exposure, its practical application is constrained by the limited availability of tissue samples. Therefore, combining measurements of AFB1-DNA and albumin adducts can effectively distinguish between recent and chronic aflatoxin exposure statuses [6].

Despite extensive research on aflatoxins and their toxicological impacts, there remains a significant knowledge gap regarding reliable biomarkers for chronic AFB1 exposure, specifically in cattle populations. Most available studies have primarily focused on feed analysis for diagnosing aflatoxicosis; however, feed-based assessments only reflect immediate contamination and fail to account for chronic, individual-level exposure variability. In addition, previous studies have largely examined aflatoxin biomarkers such as AFB1-albumin and AFB1-DNA adducts in experimental animals and human populations, with limited exploration in cattle. Consequently, there is insufficient evidence supporting the validity and practical application of these biomarkers in naturally exposed cattle populations, leaving a critical gap in the accurate assessment of long-term aflatoxin exposure in cattle and associated health risks.

This study aimed to bridge this knowledge gap by evaluating serum AFB1-albumin and blood AFB1-DNA adducts as potential biomarkers for chronic aflatoxin exposure in cattle. Specifically, this study was designed to assess the presence and quantitate the levels of these biomarkers in cattle from diverse settings – including farms, clinical cases, and slaughterhouses – to provide comprehensive insights into chronic aflatoxin exposure irrespective of immediate feed contamination levels. In addition, the study aimed to establish a novel method for synthesizing the AFB1-FAPy adduct to serve as a standard, thereby enabling accurate and sensitive quantification of AFB1-DNA adducts in cattle. Ultimately, the objective of the study is to validate the practical utility of these biomarkers to facilitate proactive and reliable monitoring of aflatoxin exposure, enhancing animal health management practices and ensuring food safety standards.

## MATERIALS AND METHODS

### Ethical approval

Written informed consent regarding the study was obtained from the cattle owners. Permission was obtained from the competent authority (Municipal Commissioner, Government of Puducherry, India) for collecting materials from the slaughterhouse at Puducherry. The study was approved under reference No.1104/PG/RIVER/2021-22 by the Rajiv Gandhi Institute of Veterinary Research and Education, Pudu-cherry, India.

### Study period and location

The study was conducted on crossbred cattle in Puducherry, India, from December 2021 to March 2022. Samples were collected from three categories of cattle: Farm-raised cattle, clinical cases from the veterinary hospital, and slaughterhouse animals.

### Chemicals

The AFB1 standard was obtained from Sigma-Aldrich (St. Louis, MO, USA). Analytical-grade solvents for mobile-phase preparations were procured from Sigma-Aldrich (St. Louis, MO, USA). All other chemicals used in the study were of analytical grade. The DNA required for the analysis was synthesized from the blood of the cattle selected for the study.

### Biosafety

Since AFB1 and its derivatives are potent carcinogens, strict biosafety measures were imple-mented to minimize personnel exposure and prevent laboratory contamination throughout the study.

### Selection and clinical examination of animals

A total of 53 cattle were selected for this study. The cattle were sourced from three categories: (a) Farm cattle from small and marginal farms in Puducherry (n = 27), (b) clinical cases from the Large Animal Medicine Unit at the Veterinary Clinical Complex, Rajiv Gandhi Institute of Veterinary Education and Research, Puducherry (n = 13), and (c) Slaughterhouse cattle from the municipal slaughterhouse in Puducherry (n = 13). Farm and clinical cases comprised of crossbred female cattle, whereas slaughterhouse samples included only male cattle. All animals were aged between 1 and 6 years and underwent a comprehensive clinical examination, including body condition scoring, rectal temperature, pulse rate, respiratory rate, conjunctival mucosa assessment, rumen motility, feeding behavior, and voiding patterns [7].

### Feed samples and aflatoxin analysis

A total of 40 feed samples were collected for aflatoxin analysis. Feed samples for the farm cattle were obtained directly from the respective farms. For the clinical cases, the feed samples were collected from the respective households. Feed analysis was not conducted for slaughterhouse cattle due to the lack of information on their dietary history. Feed samples were stored in airtight containers at temperatures below 10°C until analysis. Before analysis, the feed samples were homogenized to ensure uniformity. A portion of 250 g subsamples was drawn and subjected to quantitative analysis for AFB1 and AFB2 concentrations using a two-dimensional thin-layer chromatographic (TLC) method [8]. Briefly, toxins were extracted with acetonitrile and potassium chloride in hydrochloric acid, filtered, and defatted twice with hexane. The fat-free extract was subsequently extracted with chloroform, dried, re-diluted with chloroform, and spotted onto pre-coated silica gel G (0.25 mm thickness) on alumina sheets (Merck) alongside known standards. The plates were developed first in chloroform and acetone (9:1) in one direction, followed by toluene: ethyl acetate: formic acid (5:4:1) in the perpendicular direction. The plates were dried and examined under long-wave ultraviolet (UV) light (365 nm), and the toxin concentrations were expressed as micrograms per kilogram (μg/kg).

### Collection and analysis of blood samples

Blood samples (10 mL) were collected through jugular venipuncture from all 53 animals. Three milliliters were placed into clot activator vials for serum separation, whereas 7 mL was collected into acid citrate dextrose (ACD) tubes for further analyses.

### Estimation of serum AFB1-albumin adduct

The serum was separated from the blood by cent-rifugation and stored at −20°C until analyzed. The serum was used for quantitative analysis of the AFB1-albumin adduct using an enzyme-linked immunosorbent assay (ELISA) kit (Chongqing Biospes Co., Ltd, #BZEK1587, China) based on standard sandwich enzyme-linked immunosorbent assay technology.

Briefly, 50 μL of standard solutions (provided with the kit) and diluted serum samples were added to the corresponding microwells. Then, 100 μL of horseradish peroxidase (HRP)-conjugated anti-aflatoxin human serum albumin antibody was added to each well, excluding the control wells. The plate was manually shaken and incubated for 30 min at 37°C. Following incubation, the wells were washed 5 times with the wash buffer. Subsequently, 50 μL of TMB substrate A and 50 μL of TMB substrate B were sequentially added to each well, and the plate was incubated in the dark at 37°C for 15 min. The reaction was terminated by adding 50 μL of stop solution into each well, and absorbance was measured at 450 nm using a microplate reader (Tecan Infinite F50, Switzerland) within 15 min.

The standard curve was plotted as the relative optical density (O.D. 450 nm) of each standard solution against its corresponding concentration. The bovine AFB1-albumin adduct concentration in the samples was estimated from the standard curve. The adduct concentrations (pg AFB1-lys/mL serum) were normalized to the total serum albumin content, which was determined photometrically and expressed as pg AFB1-lys adduct per mg of albumin [9].

### Estimation of AFB1-DNA adduct in blood

#### Extraction of AFB1-DNA adduct from blood

To estimate AFB1-DNA adducts, 7 mL of blood sample was dispensed into a conical tube containing acid citrate dextrose (ACD) and centrifuged at 313 × *g* for 15 min to obtain the buffy coat. To 300 μL of the buffy coat, 950 μL of RBC lysis buffer was added, and the mixture was incubated for 10 min with intermittent mixing. After incubation, the mixture was centrifuged at 704 × *g* for 10 min. The supernatant was discarded, and the cell pellet was washed with phosphate-buffered saline (PBS).

To the pellet, 600 μL of ice-cold cell lysis buffer was added, vortexed to disperse the cells evenly, and incubated for 10 min at 37°C. Following this, 200 μL of sodium acetate was added, vortexed for 20 s, incubated at 4°C for 10 min, and centrifuged at 9,660 × *g* for 3 min. The supernatant was transferred to a fresh tube, to which 600 μL of isopropanol was added, and the mixture was centrifuged at 9,660 × *g* for 1 min. The supernatant was discarded, and the DNA pellet was washed sequentially with 600 μL of 70% ethanol followed by absolute ethanol. After centrifugation at 9,660 × *g* for 1 min, the supernatant was discarded, and the DNA pellet was air-dried.

The pellet was dissolved in 100 μL of tris-ethylen-ediaminetetraacetic acid buffer (pH 8.0), incubated at 65°C for 1 h, and stored at −20°C until further analysis. At the time of analysis, after thawing, all DNA samples were treated with 15 mM sodium carbonate (Na_2_CO_3_) and 30 mM sodium bicarbonate (NaHCO_3_) (pH 9.6) for 2 h at 37°C to ensure the conversion of AFB1-DNA adducts to their ring-opened AFB1-FAPy adduct form. The contents were subsequently neutralized, precip-itated with ethanol (95%), and stored at −80°C until analysis.

### Synthesis and characterization of aflatoxin-DNA (AFB1-FAPy adduct) standard

All reactions were performed under subdued light to minimize the formation of photoproducts. The AFB1-DNA adduct was synthesized using calf thymus DNA (Sigma: Cat No. D 3664) as the DNA source. Calf thymus DNA (20 μg) was dissolved in 2.0 mL of 20 mM sodium phosphate buffer (pH 7.4) (aqueous phase/buffer fraction) and mixed with 100 μg of AFB1 dissolved in dichloromethane (organic phase). To this two-phase system, 20 μL of a 10 mg/mL freshly prepared chloro-perbenzoic acid solution in dichloromethane was added and vigorously shaken at room temperature (28^°^C) for 24 h under dark conditions.

After incubation, the contents were cooled on ice, and the two phases were separated by centrifugation at 1,353 × *g* for 5 min. The AFB1-DNA formed was purified from the buffer fraction by extraction with an equal volume of chloroform, twice. The two phases were vigorously shaken for 20 min at 28^°^C and separated by centrifugation at 2,191 × *g* for 10 min. The AFB1-DNA was then precipitated from the buffer fraction using three volumes of cold ethanol. The DNA was pelleted by centrifugation at 9,660 × *g* for 5 min, washed twice with ethanol, and air-dried. The DNA pellets were redissolved in Tris-HCl buffer (pH 7.0) and treated with two volumes of 15 mM Na_2_CO_3_/30 mM NaHCO_3_ (pH 9.6) for 2 h to convert the AFB1-DNA adduct to the imidazole ring-opened form (AFB1-FAPy adduct). The AFB1-FAPy adduct was re-precipitated with 95% ethanol and dissolved in TE buffer (pH 8.0).

Before converting the AFB1-DNA adduct to its imidazole ring-opened form, an aliquot of the buffer and organic phases was subjected to TLC [10]. The observed zero and 0.84 retention factor (Rf) values for the buffer and organic fractions, revealed by TLC-fluorodensitometric analysis, confirmed the presence of the AFB1-N7-guanine adduct (AFB1-DNA) and unreacted AFB1 or its metabolites, respectively. In the UV range, AFB1, DNA, and the AFB1-FAPy adduct showed absorption maxima at 362 nm, 260 nm, and 360 nm, respectively [10].

The synthesized AFB1-FAPy adduct was quantified by UV absorbance at 360 nm using a Nanodrop spectrophotometer (Thermo Fisher Scientific, Waltham, MA, USA). The concentration of the AFB1-FAPy adduct was calculated using Beer–Lambert’s law: A = εlc, where ε is the molar extinction coefficient (18,000 L/mol/cm), A is the absorbance at 360 nm, l is the path length (1 cm), and c is the concentration (moles/L) [10]. Using the synthesized AFB1-FAPy adduct as the standard, the concentration of AFB1-DNA adduct in the samples was calculated as pmol of AFB1-DNA adduct per 100 ng of DNA and expressed as pmol/μg DNA.

### Estimation of AFB1-FAPy adduct in blood by ELISA

Polystyrene microtiter plates (96 wells) were coated with 100 ng of DNA samples (test wells) and serially diluted AFB1-FAPy adduct standards (standard wells) in 50 μL of coating buffer. Plates were incubated overnight at 37°C and then washed with PBS-Tween (PBST). The wells were blocked for non-specific binding with 1% bovine serum albumin (BSA) in PBST for 1 h and subsequently washed with a washing buffer.

Following blocking, antisera (anti-AFB1-DNA adduct antibody, Novus Biologicals, USA) diluted at 1:5000 (100 μL/well) in PBST with 0.01% BSA was added to the wells. Plates were incubated for 2 h at 37°C before washing with PBST to remove unbound antibodies. After washing, 100 μL/well of a 1:1000 dilution of HRP-conjugated anti-mouse IgG raised in goats (Sigma-Aldrich, St. Louis, MO, USA) was added. The plates were incubated at 37°C for 1 h and then washed.

Subsequently, 100 μL of freshly prepared chro-mogenic substrate solution containing o-phenylenedi-amine dihydrochloride was added to each well, and the plates were incubated at 28^°^C for 15 min. The enzyme-substrate reaction was stopped by adding 100 μL of stop solution (1 M sulfuric acid) per well. The O.D. was recorded at 490 nm using an ELISA Reader (Thermo Fisher Scientific).

A standard curve was constructed by plotting the relative O.D. 490 nm of serially diluted AFB1-DNA adduct standards against their respective concentrations. The AFB1-DNA adduct concentration in the test samples was estimated from the standard curve and expressed as pmol/μg DNA.

### Statistical analysis

AFB1-albumin and AFB1-DNA adduct concentra-tions were analyzed using non-parametric statistical methods [11] in the Statistical Package for the Social Sciences (version 26.0, SPSS Inc., Chicago, IL, USA). The Kruskal–Wallis H test with Dunn’s multiple comparisons was used to determine statistical significance. A p < 0.05 was considered statistically significant. Spearman’s rank correlation test was employed to assess the relationship between AFB1-albumin and AFB1-DNA adduct levels.

## RESULTS

### Clinical findings

The body condition scores (BCS) of the various categories of cattle, assessed based on a 0–6 point scale, indicated that among the farm cattle, 19 animals (70.37%) had a BCS of 3, followed by a BCS of 2 in 22.22%, and a BCS of 4 in 7.41%. Within the clinical cases of anorexia, an equal proportion of animals, 6 (46.15%), had a BCS of 2 and 3, respectively, followed by 1 animal (7.7%) with a BCS of 1. Among the slaughtered cattle, 11 animals (84.62%) had a BCS of 3, and 2 animals (15.38%) had a BCS of 2.

All animals included in the study exhibited normal body temperatures, and no significant differe-nces were observed between the three categories. Most animals presented with pink conjunctival mucous membranes, indicating healthy systemic status. Rumen contractions were normal in most cattle. Among the farm cattle, 88% exhibited a normal appetite. However, rumen contractions were decreased in six animals (four clinical cases and two farm cattle) and sluggish in six animals (three each from farm and clinical cases). The majority of animals in the farm and clinical groups had normal voiding habits. Nevertheless, seven farm animals (25.93%) exhibited diarrhea, whereas four clinical cases (30.77%) showed constipation. Solitary cases of melena and semi-solid feces were also recorded.

### Analysis of aflatoxin in feed samples

The analysis of feed samples (only for farm cattle and clinical cases) indicated that 50% of the samples were positive for aflatoxins AFB1 and AFB2 (Table 1). Among these, 70% exceeded the maximum permissible limit (MPL) of aflatoxin B1 (20 μg/kg) recommended by the Bureau of Indian Standards [12].

**Table 1:** Aflatoxin concentration (μg/kg) in cattle feed samples.

Source of sample	No. of samples +ve in AFB1	Level of AFB1 (µg/kg) (Mean ± SD)	Range (µg/kg)	No. of samples +ve in AFB_2_	Level of AFB2 (µg/kg) (Mean ± SD)	Range (µg/kg)	Samples with aflatoxin levels exceeding the MPL
Farms (n = 27)	14 (51.85%)	28 ± 26.23	(7–87)	4 (14.81%)	10.5 ± 4.04	(7–14)	10 (71.43%)
Clinical cases) (n = 13)	6 (46.15%)	128 ± 171.68	(7–464)	4 (30.77%)	47 ± 46.54	(14–116)	4 (66.67%)

AFB1=Aflatoxin B1, AFB2=Aflatoxin B2, MPL=Maximum permissible limit (20 μg/kg, BIS, 2009), SD=Standard deviation

Specifically, among the feed samples collected from farms, 51.85% tested positive for aflatoxins, and within these positive samples, 71.43% had aflatoxin concentrations above the MPL. Similarly, in the feed samples from clinical cases, 46.15% were positive for aflatoxin, and 66.67% of these had aflatoxin contents exceeding the MPL.

### Serum AFB1-albumin adduct analysis

The concentrations of AFB1-albumin adducts in the serum of different categories of cattle, as deter-mined by ELISA, are presented in Table 2.

**Table 2:** AFB1-albumin adduct in the serum of cattle.

Category of animals	AFB1-albumin adduct (pg/mg albumin)			

	Median	Percentile		p-value

		Q1	Q3	
Farm cattle (n = 27)	0.730	0.350	0.790	0.731
Clinical cases (n = 13)	0.670	0.445	0.930	
Slaughterhouse cattle (n = 11)	0.770	0.550	0.840	

Data were analyzed using the Kruskal–Wallis test with Dunn’s multiple comparisons test. AFB1=Aflatoxin B1

As shown in Table 2, AFB1-albumin adducts were detected in serum samples across all three categories of cattle. The Kruskal–Wallis H test indicated no significant differences in the dependent variable among the different categories, χ^2^(2) = 0.63, p = 0.731, with a mean rank score of 24.46 for farm cattle, 27.42 for clinical cases, and 28.09 for slaughterhouse cattle. The median levels of AFB1-albumin adducts were nearly similar among all three categories. Furthermore, no significant differences were observed in AFB1-albumin adduct levels between different breeds, age groups, or sexes of the cattle (data not shown).

The serum AFB1-albumin adduct levels suggested that all animals, irrespective of their category, were chronically exposed to aflatoxin-contaminated diets, thereby indicating long-term exposure.

### Analysis of serum AFB1-FAPy adduct content by ELISA

#### Detection and quantification of AFB1-FAPy adduct

**Figure 1:**
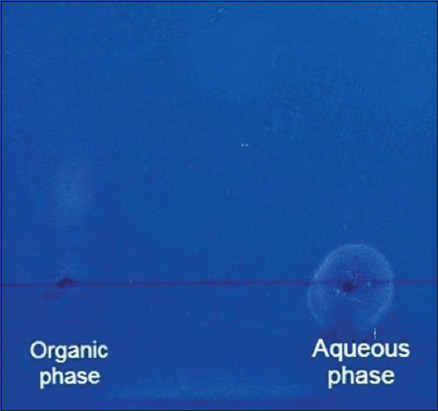
Thin-layer chromatography image (fluores-cence 365 nm) shows the localization of aflatoxin B1-formamidopyrimidine adduct with retention factor=0 (Aqueous phase).

AFB1-DNA adduct (AFB1-FAPy) standards was successfully synthesized and characterized. TLC analysis of the buffer fraction (aqueous phase) of the reaction mixture revealed a single fluorescent spot under 365 nm UV light with an Rf value of zero, confirming the presence of the AFB1-FAPy adduct (Figure 1).

#### Estimation of AFB1-DNA adduct in blood samples

The concentration of AFB1-DNA adduct was meas-ured in the blood samples of the cattle (Table 3).

**Table 3:** AFB1-DNA adduct (pmol/μg DNA) in the blood of cattle.

Category of animals	AFB1-DNA adduct (pmol/µg DNA)			

	Median	Percentile		p-value

		Q1	Q3	
Farm cattle (n = 23)	14.76	10.33	26.67	0.328
Clinical cases (n = 12)	7.47	3.85	23.62	
Slaughterhouse cattle (n = 11)	18.33	7.34	28.64	

Data were analyzed using the Kruskal–Wallis test with Dunn’s multiple comparisons test. AFB1=Aflatoxin B1

AFB1-DNA adducts were detected in all samples, irrespective of their category. The Kruskal–Wallis H test revealed no significant differences in the dependent variable among the different categories, χ^2^(2) = 2.23, p = 0.328, with a mean rank score of 25.59 for farm cattle, 18.58 for clinical cases, and 24.5 for slaughterhouse cattle. The median levels of AFB1-DNA adduct in the blood of farm and slaughterhouse cattle were numerically higher compared to those in clinical cases.

### Correlation between blood levels of AFB1-DNA adduct and AFB1-albumin adduct, and AFB1 levels in feed

Correlations were evaluated between aflatoxin DNA adducts and albumin adducts in farm, clinical, and slaughterhouse cases, as well as between the adducts and the AFB1 levels in the feed of farm and clinical cases. Spearman’s rank correlation test was employed to assess the relationship between AFB1-albumin and AFB1-DNA adduct levels.

The results of the Spearman correlation analysis indicated a non-significant, small, positive relationship between the aflatoxin DNA adduct and albumin adduct in farm cattle (r[23] = 0.209, p = 0.339); a non-significant, negative relationship in clinical cases (r[12] = −0.189, p = 0.606); and a non-significant, medium positive relationship in slaughterhouse cattle (r[11] = 0.355, p = 0.284).

In addition, there was a small negative correlation between the levels of AFB1 in the feed and the serum AFB1-albumin adduct (r[40] = −0.104, p = 0.524), as well as between feed AFB1 levels and the AFB1-DNA adduct concentrations (r[35] = −0.220, p = 0.205).

To further assess whether adduct levels in the blood of cattle were associated with aflatoxin levels in the feed, a comparative analysis between the variables was conducted (Table 4).

**Table 4:** Comparison of the levels of AFB1 in feed and the levels of AFB1 adducts in the blood of cattle.

Percentage of samples	AFB1 levels in feed (µg/kg)	Mean AFB1-albumin adduct (pg/mg albumin)	Mean AFB1-DNA adduct (pmol/µg DNA)
50	0	0.68	7.74
15	7	0.77	15.74
15	22	0.55	5.26
5	29	0.42	2.59
2.5	58	0.43	4.59
5	87	0.73	4.95
5	116	0.50	12.44
2.5	464	0.98	32.45

AFB1=Aflatoxin B1

The AFB1 concentrations in the feed samples ranged from 0 to 464 μg/kg. The mean levels of albumin adduct and DNA adduct varied from 0.42 to 0.98 pg/mg albumin and from 2.59 to 32.45 pmol/μg DNA, respectively. As evident from Table 4, even though AFB1 levels were zero in 50% of the feed samples, moderate levels of AFB1-albumin adducts and AFB1-DNA adducts were still detected in the blood of the cattle that consumed the feed.

## DISCUSSION

Aflatoxins are regarded as the most important mycotoxins due to the wide range of food commodities they can contaminate, their persistence in food prod-ucts once formed, and their potent carcinogenic prop-erties. Although aflatoxin contamination was previously considered a concern primarily in tropical and subtr-opical regions, climate change and international trade have now elevated its importance even in temperate countries [2].

This study analyzed 40 feed samples from farm cattle and anorexic clinical cases for aflatoxins B1 and B2. Among these, 50% of the samples tested positive for aflatoxins, with 70% exceeding the MPL of AFB1 (20 μg/kg) set for dairy cow feed [12]. These findings underscore a high level of cattle feed contamination in the Puducherry region. Previous surveys by Sarath-chandra and Muralimanohar [13], Umaya *et al*. [14], Mohanamba *et al*. [15] conducted across southern India reported aflatoxin concentrations ranging from 1.8 to 540 μg/kg in livestock feeds.

Groundnut oil cake was identified as a major ingredient in total mixed rations in the present study and likely contributed to elevated aflatoxin levels. A previous study by Mohanamba *et al*. [15] has shown that groundnut cake samples exhibit higher aflatoxin contamination compared to wheat bran, rice polish, and rice bran. Even low levels of aflatoxin exposure are detrimental to animal health, affecting biochemical and hematological parameters [16, 17].

The toxicity of AFB1 is mediated through bioacti-vation and detoxification pathways within the animal. AFB1, as a pro-carcinogen is bioactivated by cytochrome P450 enzymes to produce the highly reactive AFB1-8,9-epoxide [5]. This electrophilic metabolite spontaneously binds to cellular macromolecules, such as DNA and proteins, forming AFB1-DNA and AFB1-albumin adducts, respectively. These adducts are considered robust biomarkers of AFB1 exposure, reflecting the amount of AFB1 bioactivated and available to exert deleterious effects [5, 6].

Protein adduct measurements, particularly those involving albumin, provide valuable insights into chronic aflatoxin exposure [5]. Albumin, the major serum protein synthesized exclusively in the liver, binds covalently with AFB1; it is estimated that 1%–3% of an AFB1 dose binds to albumin in experimental animals [6]. In this study, AFB1-albumin adducts were detected in all cattle serum samples, indicating regular ingestion of aflatoxin-contaminated diets and confirming long-term exposure. The median AFB1-albumin adduct concentration obse-rved was 0.73 pg/mg albumin (interquartile range: 0.45–0.83 pg/mg). A previous study by Srinivas [18] has reported higher mean AFB1-albumin adduct levels, such as 6.25 ng/mg albumin in Indian cattle, and a range from below detection limits to 96.3 pg/mg albumin (median: 20.3 pg/mg albumin) in Kenyan cattle [19]. Although the concentrations reported in the present study are comparatively lower, they are still indicative of chronic exposure.

Human epidemiological studies indicate that the more often an aflatoxin-contaminated diet is consumed, the higher the AFB_1_-albumin adduct level in the blood [20]. Detection of AFB1-albumin adducts is a valuable biomarker because albumin reflects hepatocyte exposure and liver damage [21]. Further, a single measurement integrates aflatoxin exposure from all routes, accounting for individual variability in metabolism and clearance [3, 22]. Therefore, albumin adduct levels serve as a reliable indicator of long-term exposure.

Similarly, AFB1-DNA adducts provide a measure of the biologically effective dose of AFB1 [23]. Human studies have detected AFB1-DNA adducts in tissue samples such as the liver, placenta [23], and peripheral blood leukocytes in high-exposure regions [24]. The major AFB1-DNA adduct forms when AFB1-8,9-epoxide binds to the N7 position of guanine, which can subsequently open to form the more stable AFB1-FAPy adduct [10, 25]. Monoclonal antibody 6A10 has been demonstrated to effectively detect the AFB1-FAPy adduct in humans and animals [25, 26].

Prior to the present study, there were no published reports on the quantification of AFB1-FAPy adducts in the blood of cattle. Given the widespread aflatoxin exposure in cattle, quantifying AFB1-DNA adducts in blood is of significant molecular epidemiological value. In this study, a novel method was developed to synthesize AFB1-FAPy adducts using calf thymus DNA, and the presence of AFB1-FAPy adducts in cattle blood was successfully detected using an indirect ELISA.

AFB1-DNA adducts were detected in all blood samples, regardless of category. Notably, AFB1-DNA and AFB1-albumin adducts were present even in cattle whose feed showed undetectable aflatoxin levels. The overall median level of AFB1-DNA adducts was 12.91 pmol/μg DNA (interquartile range: 8.22–24.53 pmol/μg DNA). Due to the absence of similar studies in cattle, direct comparisons are not possible. However, in humans, 57.5% of placental samples in a high-AFB1 exposure area contained AFB1-DNA adducts, with concentrations ranging from 0.6 to 6.3 μmol/mol DNA [26].

Globally, over 80% of hepatocellular carcinoma (HCC) cases occur in developing countries, where AFB1 is a major etiological factor [27]. A case–control study including 380 HCC patients and 588 controls revealed significantly higher AFB1-DNA adduct levels in HCC cases compared to controls (2.01 ± 0.71 vs. 0.98 ± 0.63 μmol/μg DNA) [28].

Previous research suggests that AFB1-albumin adduct levels in serum correlate with AFB1-DNA adduct formation in the liver. Studies by Wild [6] in rats demonstrated a constant relationship between aflatoxin binding to plasma albumin and liver DNA after both single and multiple doses of AFB1. In the present study, correlation analysis between AFB1-albumin and AFB1-DNA adducts revealed a moderate correlation (r = 0.6, p = 0.609). Several studies reviewed by Kensler and Eaton [5] have similarly reported positive associations between aflatoxin-DNA and albumin adduct levels in human populations exposed to contaminated food. However, differences in adduct kinetics, metabolic pathways, and genetic factors may influence the strength of the correlation between aflatoxin-DNA and albumin adducts. Thus, although these biomarkers are correlated, they should be interpreted cautiously and in conjunction with additional exposure and health data.

Despite the detection of aflatoxins in feed and blood samples in this study, no overt toxicological manifestations were observed clinically. Body condition scoring, which can reflect chronic health impacts, showed that the majority of cattle had average scores (BCS 3), and no significant differences were found among the various categories. Clinical manifestations of aflatoxicosis depend on the dose and duration of exposure [2, 29].

Aflatoxin-contaminated feeds not only threaten livestock health and productivity [2] but also pose indirect risks to human health through contaminated animal products such as milk and meat [30–32].

## CONCLUSION

This study demonstrated a significant prevalence of aflatoxin contamination in cattle feed samples from Puducherry, with 50% of the tested feeds positive for aflatoxins and 70% exceeding the maximum permissible limit of 20 μg/kg. Biomarker analysis revealed the consistent presence of AFB1-albumin adducts and AFB1-DNA adducts in the blood of cattle across farm-raised, clinical, and slaughterhouse categories. Notably, even in animals consuming feed samples with undetectable aflatoxin levels, moderate levels of both biomarkers were detected, highlighting the persistent and cumulative nature of aflatoxin exposure.

The practical implication of these findings is that routine feed testing alone may not accurately reflect chronic exposure risks, underscoring the critical importance of biomonitoring through valid-ated biomarkers such as serum AFB1-albumin and AFB1-DNA adducts. The detection of these biomarkers offers a sensitive and integrative approach for assessing long-term aflatoxin exposure in cattle populations, which is crucial for animal health management and for minimizing potential aflatoxin transfer to human consumers through milk and meat products.

A major strength of this study is the novel development and successful application of an indirect ELISA method for the detection of AFB1-FAPy adducts in bovine blood, marking a significant advancement in molecular epidemiological surveillance for aflatoxin exposure in livestock. Furthermore, this study is among the first to document the simultaneous detection of both AFB1-DNA and AFB1-albumin adducts in cattle under field conditions in India.

However, the study is limited by its relatively small sample size and cross-sectional design, which captured only a single time-point exposure profile. Variability in feed intake, individual metabolic responses, and the lack of longitudinal monitoring could have influenced the levels of biomarkers observed.

Future research should focus on longitudinal cohort studies to monitor dynamic changes in aflatoxin biomarkers over time and to better establish dose-response relationships between feed contamination levels, biomarker burden, and clinical outcomes. In addition, the establishment of standardized refe-rence ranges for AFB1-albumin and AFB1-DNA addu-cts in cattle would further enhance the utility of these biomarkers for routine surveillance programs. Integrating biomarker analysis with feed testing would offer a comprehensive strategy to safeguard animal health, optimize productivity, and ensure food safety in livestock production systems.

## AUTHORS’ CONTRIBUTIONS

PP: Designed the study, investigation, analyzed data, and wrote -results and original draft. USR: Designed the study, investigation, collected data, compiled results, and drafted the manuscript. MGN: Conceptualized and supervised the study, project admi-nistered, and edited the manuscript. US: Methodology, formal analysis, and writing–editing of the manuscript. JT, RK, and AWL: Planned the study, provided methodology inputs, and drafted and revised the manuscript. VP: Conceptualized, reviewed clinical cases, and sample collection and edited the manuscript. PS: Sample analysis, data analysis, and editing of the manuscript. All authors have read and approved the final manuscript.
